# *PITX2*‐dependent gene regulation in atrial fibrillation and rhythm control

**DOI:** 10.1113/JP273123

**Published:** 2017-04-25

**Authors:** Fahima Syeda, Paulus Kirchhof, Larissa Fabritz

**Affiliations:** ^1^ Institute of Cardiovascular SciencesUniversity of BirminghamBirminghamUK; ^2^ Department of CardiologyUHB NHS TrustBirminghamUK; ^3^ Department of CardiologySWBTBirminghamUK; ^4^ Department of Cardiovascular Medicine, Division of RhythmologyUniversity Hospital MünsterMünsterGermany

**Keywords:** antiarrhythmic drugs, atrial fibrillation, gene regulation, mouse model, Pitx2, transcription factors

## Abstract

Atrial fibrillation (AF) is a common arrhythmia. Better prevention and treatment of AF are needed to reduce AF‐associated morbidity and mortality. There are several major mechanisms that cause AF in patients, including a genetic predisposition to develop AF. Genome‐wide association studies have identified genetic variants associated with AF populations, with the strongest hits clustering on chromosome 4q25, close to the gene for the homeobox transcription factor PITX2. The effect of these common gene variants on cardiac *PITX2* mRNA is currently under study. PITX2 protein regulates right–left differentiation of the embryonic heart, thorax and aorta. PITX2 is expressed in the adult left atrium, but much less so in other heart chambers. *Pitx2* deficiency results in electrical and structural remodelling, and impaired repair of the heart in murine models, all of which may influence AF through divergent mechanisms. *PITX2* levels and single nucleotide polymorphisms on chromosome 4q25 may also be a predictor of the effectiveness of anti‐arrhythmic drug therapy.

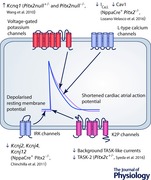

**Abbreviations**
AF: atrial fibrillation
GWAS: genome‐wide association studies
miRNA: microRNA
PITX: paired‐like homeodomain transcription factor human and murine protein
*PITX*: paired‐like homeodomain transcription factor human gene
*Pitx*: paired‐like homeodomain transcription factor murine gene
SNP: single nucleotide polymorphism

## Introduction

Atrial fibrillation (AF) affects 2–3% of the population in Europe and the US and the prevalence of AF is rising (Schnabel *et al*. 2015; Kirchhof *et al*. 2016). AF is a significant cause of death, stroke, dementia and reduced quality of life in the Western world and this issue is complicated by a lack of effective therapies. The treatment of AF has four different domains: treatment of underlying cardiovascular conditions, stroke prevention, rate control and rhythm control. Even on optimal stroke prevention and rate control therapy, cardiovascular morbidity and mortality remains high (Marijon *et al*. 2013; Bassand *et al*. 2016). The success of rhythm control therapy is often unpredictable and recurrence is common. This outcome is partly attributable to our limited understanding of the underlying genetic causes of AF and the interaction of these causes with type of rhythm control therapy. Initial observations suggest that the haplotype of common AF‐related variants modifies the outcome of anti‐arrhythmic therapy (Parvez *et al*. 2012; Huang & Darbar, 2016), suggesting that rhythm control therapy could benefit from precision and personalisation based on genomic information.

## AF has a heritable component

AF appears to be familial in approximately 15% of early‐onset AF without concomitant diseases and 5% of all AF cases (Darbar *et al*. 2003), and a family history of early‐onset AF increases overall AF risk (Fox *et al*. 2004). Linkage and functional studies have revealed the mutation of several potassium channels such as KCNQ1, KCNE2, KCNH2 in rare, monogenic families with a Mendelian pattern of AF inheritance (Chen *et al*. 2003; Yang *et al*. 2004; Hong *et al*. 2005; Xia *et al*. 2005). These gain or loss of function potassium channel mutations may account for some of the electrophysiological changes that promote AF, such as reduced wavelength or early after‐depolarisations, respectively. However, most AF patients do not harbour these rare genotypes with clear phenotypes, so the polygenic and multifactorial nature of AF is an important avenue of research. Therefore, transcriptional regulators with multiple effectors may play a significant role in familial AF caused by common genetic variants.

## Common gene variants associated with AF

Genome‐wide association studies (GWAS), unbiased correlation studies designed to identify associations between allele frequencies and trait variation, have identified multiple loci that associate with AF (Gudbjartsson *et al*. 2007; Benjamin *et al*. 2009; Kaab *et al*. 2009; Ellinor *et al*. 2010, 2012; Schnabel *et al*. 2011). Initial GWAS identified two single nucleotide polymorphisms (SNPs; rs2200733 and rs10033464) in European and Chinese populations (Gudbjartsson *et al*. 2007) on chromosome 4q25. Other loci exist on chromosome 16q22 within intron 1 of the gene encoding ZHFX3, i.e. zinc finger homeobox protein 3 (Benjamin *et al*. 2009) and on chromosome 1q21 on intron 1 of the gene for KCNN3 (involved in calcium‐activated potassium channels; Ellinor *et al*. 2010). Since these initial studies, a multitude of SNPs that associate with AF have been identified on chromosome 4q25. The gene variants on chromosome 4q25 are intergenic, but many of them are in a genomic ‘desert’ approximately 150 kb upstream from the gene for PITX2 (paired‐like homeodomain transcription factor).

## The PITX gene family

The PITX family of homeobox transcription factors consists of PITX1, PITX2 and PITX3, each of which has distinct and overlapping expression patterns, therefore functions, in different organs (Cox *et al*. 2002). All three members of the PITX family, but predominantly PITX2 and PITX3, are expressed in the anterior segment of the eye. In humans, PITX2 mutations are associated with Axenfeld‐Rieger syndrome and PITX3 mutations with congenital cataracts (Semina *et al*. 1998). PITX1 is also essential for hindlimb and pituitary development (Szeto *et al*. 1999), and PITX2 for tooth, heart, lung and abdominal development in the mouse (Lin *et al*. 1999).

Three PITX2 isoforms (PITX2a, PITX2b and PITX2c), which are generated by alternative splicing and differential promoter usage of the PITX2 gene, are highly expressed in mice and humans during development (Schweickert *et al*. 2000). The PITX2d isoform, which exists in humans only, suppresses the transcriptional activity of the PITX2a and PITX2c isoforms (Cox *et al*. 2002). The predominant cardiac isoform of PITX2 is PITX2c (Kirchhof *et al*. 2011).

## PITX2 promotes left–right asymmetry

PITX2 was initially described in the context of embryonic development of left–right asymmetry of internal organs: it is expressed in the left heart and gut of the mouse, chick and *Xenopus*, and its misexpression alters position and the twisting of organs (Ryan *et al*. 1998; Lin *et al*. 1999; Campione *et al*. 2001).

The cardiac system shows left–right asymmetry, e.g. normal coordinated heartbeat is generated from the sinoatrial nodal pacemaker cells in the right atrium. The development of cardiac left–right specific characteristics such as the restriction of the sinoatrial node to the right atrium is critically dependent on asymmetrical organ morphogenesis (Mommersteeg *et al*. 2007; Galli *et al*. 2008). Cardiac left–right asymmetry is subject to left‐sided PITX2 expression (Galli *et al*. 2008; Tessari *et al*. 2008), particularly the PITX2c isoform (Schweickert *et al*. 2000; Kirchhof *et al*. 2011) through the lefty–nodal programming pathway (Wang *et al*. 2010).

## PITX2‐dependent gene expression in the adult heart

Triggered by the GWAS study pointing to a possible role for PITX2 in AF, the role of PITX2 in the adult heart, in addition to its role in left–right asymmetry and cardiac development, has been investigated: *Pitx2c* expression continues in the postnatal left atrium in mice and humans (Wang *et al*. 2010; Kahr *et al*. 2011; Kirchhof *et al*. 2011) and there is progressive loss of *Pitx2c* with age in mice (Wang *et al*. 2010). Thus, *Pitx2c* dysregulation has the potential to influence AF in adulthood and senescence.

In AF, fast irregular atrial beats overtake the sinoatrial node, sometimes also resulting in ventricular arrhythmias. The origins of ectopic electrical activity are often in the pulmonary veins (Haissaguerre *et al*. 1998; Po *et al*. 2005) and if from the left atrium, then predominantly from the left atrial posterior wall (Sanders *et al*. 2005; Holmes *et al*. 2016). Given the crucial role of PITX2 in left–right asymmetry, it is probable that loss of PITX2 in some cases of heritable AF causes incomplete suppression of pacemaker activity in the left heart. This has been shown to be mediated by the loss of Shox2‐silencing and increase in *Hcn4* (Wang *et al*. 2010).

## PITX2 mRNA concentrations regulate atrial function

Both under‐ and overexpression of PITX2 has been found to be associated with AF (Chinchilla *et al*. 2011; Perez‐Hernandez *et al*. 2016) in humans. The variability of *PITX2* in AF patients suggests that there is a critical level of PITX2 for normal atrial function in the adult. We have recently shown a *PITX2* mRNA gradient in AF patients requiring AF ablation. Hence, AF patients could be categorised according to *PITX2* levels (Syeda *et al*. 2016). The mechanisms of AF promotion are diverse, and there are multiple pathways by which PITX2 could regulate arrhythmogenesis. This is unsurprising given that PITX2 is a transcription factor with multiple targets (Hjalt & Semina, 2005). Reduced *Pitx2* expression was associated with higher susceptibility to inducible atrial arrhythmias including AF in mice as observed by several groups (Wang *et al*. 2010; Chinchilla *et al*. 2011; Kirchhof *et al*. 2011). Shortened atrial action potential durations, a phenomenon facilitating re‐entry, were associated with *Pitx2* deficiency (Kirchhof *et al*. 2011).

Postnatal conditional deletion of all *Pitx2* isoforms in the left atrium (*Pitx2* CKO; achieved by using the muscle creatine kinase‐Cre driver) generally resulted in upregulation of genes signifying that, on the whole, PITX2 represses translation. The *Pitx2* CKO mouse had irregular resting heart rates and low amplitude P waves (Tao *et al*. 2014). The upregulation of genes associated with structural remodelling (e.g. integrin α 3 and 5) and cell‐junction assembly (e.g. desmoplakin and connexin43) indicates that loss of *Pitx2* may cause structural remodelling and damage to the intercalated disc (Tao *et al*. 2014). Prenatal atrial‐specific deletion of all *Pitx2* isoforms achieved by using a Nppa‐Cre driver (*NppaCre* ^+^ *Pitx2* ^−/−^) results in modest atrial enlargement and wall thinning during embryonic development (Chinchilla *et al*. 2011). Heterozygous deletion of isoform c‐specific exon 4 resulting in a 40% reduced left atrial *Pitx2c* expression (*Pitx2c* ^+/−^), in contrast, did not cause any obvious structural abnormalities (Kirchhof *et al*. 2011).

*Pitx2* overexpression can promote repair after myocardial injury. So it appears that there is a dynamic *Pitx2* response to stress and metabolic changes (Tao *et al*. 2016). *Cis*‐regulatory elements for the transcription factor Tbx5 in regions analogous to the human *PITX2* risk locus have also been found in the mouse. Postnatal deletion of *Tbx5* led to *Pitx2* reduction and caused atrial arrhythmias by action potential duration prolongation (Nadadur *et al*. 2016). Interestingly, an inverse relationship between the effects of *Tbx5* on some AF‐relevant ion channel expression and the effects of *Pitx2* on the same ion channels was observed (Tao *et al*. 2014) and the loss of *Pitx2* reversed the pro‐arrhythmic effects of the loss of *Tbx5* because the loss of either facilitated AF through opposite mechanisms (Nadadur *et al*. 2016).

Based on several animal models of *Pitx2* loss, where haploinsufficiency of *Pitx2* has resulted in a less severe phenotype than complete deletion, it can be deduced that there is a dose‐dependent regulation of atrial function by *Pitx2* in the adult left atrium (Wang *et al*. 2010; Kirchhof *et al*. 2011; Lozano‐Velasco *et al*. 2016).

## PITX2‐dependent ion channel regulation

Several potassium channel (Wang *et al*. 2010; Chinchilla *et al*. 2011; Kirchhof *et al*. 2011; Syeda *et al*. 2016) and calcium handling genes (Tao *et al*. 2014; Lozano‐Velasco *et al*. 2016) are regulated by *Pitx2*, as seen in mutant *Pitx2* models (see Abstract figure). The action potential duration shortening observed in *Pitx2c^+/−^* mice (Kirchhof *et al*. 2011) and depolarised resting membrane potential in NppaCre^+^Pitx2^−/−^ mice and *Pitx2c^+/−^* mice suggests that *Pitx2* regulates the expression of several potassium channels contributing to atrial repolarisation and to the resting membrane potential. Indeed, in NppaCre^+^Pitx2^−/−^, expression of *Kcnj2* is decreased (Chinchilla *et al*. 2011), which would cause a decrease in inward rectifier potassium ion channel (*I*_K1_), the primary determinant of the resting membrane potential. In *Pitx2c^+/^* ^−^ mice, both the expression of TWIK‐related acid‐sensitive K^+^ channel (TASK‐2) and TASK‐like background currents, contributors to the resting membrane potential, were reduced, though *I*_K1_ was not altered (Syeda *et al*. 2016).

## Non‐protein targets of PITX2

Multiple microRNAs (miRNAs), short non‐coding strands of RNA that usually induce post‐transcriptional gene‐silencing and fine‐tune gene signalling during tissue development and homeostatic control (Beermann *et al*. 2016), are downstream from *Pitx2* and involved in AF pathogenesis. *Pitx2* expression co‐localises with miR‐17‐92 cluster expression, and loss of *Pitx2* results in loss of multiple miRNAs that are encoded by miR‐17‐92 and its closely related homologue miR‐106b‐25. Mice deficient in these miRNA clusters share similar characteristics to *Pitx2*‐deficient mice including induced arrhythmia susceptibility and dysregulation of Shox2 and Tbx3 (Wang *et al*. 2014). The multiple miRNAs regulated by *Pitx2* may partly explain how *Pitx2* modulates several pathways potentially leading to AF (Li *et al*. 2016).

## Interactions between SNPs and transcription factors

Several genes that are either associated with AF in patients who have common intronic or distal variants shown by GWAS or rare variants that directly cause AF as shown by linkage analysis, interact with PITX2. These include genes for TBX5 (Huang *et al*. 2015; Ma *et al*. 2016; Nadadur *et al*. 2016), HCN4 (Wang *et al*. 2010; Mahida & Ellinor, 2012), KCNN3 (Ellinor *et al*. 2010; Mahida & Ellinor, 2012; Lozano‐Velasco *et al*. 2016), KCNJ2 (Xia *et al*. 2005; Chinchilla *et al*. 2011), CAV‐1 (Mahida & Ellinor, 2012; Lozano‐Velasco *et al*. 2016) and KCNQ1 (Chen *et al*. 2003; Wang *et al*. 2010), as seen in murine models. Thus, PITX2 potentially regulates AF through several genes already implicated in AF.

It has also been observed that SNPs on different AF susceptibility loci (e.g. rs2200733 on chromosome 4q25 and rs2106261 on chromosome 16q22) interact with each other in AF (Huang *et al*. 2015) and Zfhx3 has been identified as a possible target for *Pitx2* through CHIP‐Seq analysis (Tao *et al*. 2014). Furthermore, the expression of *Pitx2c* mRNA positively correlates with *ZFHX3* mRNA expression through miR‐1 (Huang *et al*. 2015).

Chromosome conformation capture studies have shown that there is long‐range interaction between the risk locus at 4q25 and the *PITX2c* promoter (Aguirre *et al*. 2015), but beyond this finding, there is little information on how variants distal to *PITX2* interact with *PITX2* to cause AF. Interestingly, an AF‐associated SNP proximal to *PITX2* has been shown to regulate *PITX2c* expression in human stem cell‐derived cardiomyocytes by regulating *PITX2* enhancer activity (Ye *et al*. 2016).

Though the interaction between genetic variants close to *PITX2* and *PITX2* itself is not completely understood, the effects of the loss of *PITX2* appear to converge with the presence of AF‐associated SNPs.

## The potential to use PITX2 to personalise AF therapy

Clinical observational studies have suggested that common AF risk alleles at chromosome 4q25 near *PITX2* modify response to anti‐arrhythmic therapy in patients (Parvez *et al*. 2012). Low *Pitx2* mRNA also improved the effectiveness of sodium channel blockers in a prospective experimental study (Syeda *et al*. 2016). *PITX2* levels vary in AF patients (Syeda *et al*. 2016) and given the observation that loss of left atrial *Pitx2* facilitates AF, it may be desirable to target those AF patients who have low *PITX2* as a distinct population for therapy. There is, however, no clear relation between atrial tissue *PITX2* mRNA levels and SNP haplotype of the common gene variants associated with AF in patients (Gore‐Panter *et al*. 2014, 2016; Syeda *et al*. 2016).

The current limited success of rhythm control therapy is thought to be due to heterogeneous drivers causing recurrent AF and modulating treatment response (Fabritz *et al*. 2016). Amongst the plethora of putative downstream targets of *Pitx2*, the resting membrane potential (Chinchilla *et al*. 2011; Syeda *et al*. 2016) is one that has been seen to be a good predictor of the sodium channel blocking effects of flecainide in isolated cells of human and mouse origin (Syeda *et al*. 2016). The resting membrane potential is also a good predictor of flecainide's anti‐arrhythmic effectiveness. By using a more precisely targeted approach, these observations of PITX2‐dependent effects may help improve rhythm therapy in the future.

## Open questions regarding research into PITX2

(1) A robust method for identification of patients with high and low atrial *PITX2* levels is needed. Clearly, measuring *PITX2* expression in atrial tissue of people who are not candidates for surgery is impracticable, so surrogate blood biomarkers of *PITX* could be used to subtype AF patient populations to bring about much‐needed leaps in personalised predictions of both AF risk and response to therapy. Currently, the P wave in the ECG still remains the best biomarker for AF (Fabritz, 2016).
(2) Well‐designed clinical trials to assess the *PITX2* dependence of the effectiveness of rhythm control therapy could help to re‐evaluate anti‐arrhythmic drugs that have often been ineffective thus far. It is likely that these findings are not limited to *PITX2* and the assessment of genotype dependence on the effectiveness of other anti‐arrhythmic drugs could be approached in this manner.
(3) Further exploration of the relevance of the resting membrane potential for anti‐arrhythmic drug therapy success in patients could be valuable.
(4) Determination of the major clinical types of AF reflecting different drivers of the arrhythmia, e.g. ‘early onset AF’ as a proxy for genetic predisposition to AF.
(5) Characterisation of the interaction of atrial stressors that are often concurrent with AF, with different *PITX2* expression levels could also guide the choice of anti‐arrhythmic drug.

## Conclusions

Although the entire scope of the activities and interactions of PITX2 are yet to be elucidated, it is clear that *PITX2* has important functions in the adult left atrium and there is evidence in animal models that reduced *Pitx2* mRNA levels predispose atria to AF by changing its electrical function, whether by abnormal pacemaker activity or adverse electrical remodelling. While complete deletion of *Pitx2* results in structural abnormalities, moderate reduction in atrial *Pitx2* levels primarily alters electrical function of the atria, for example the resting membrane potential and ion channel function.

Apparently, AF‐associated SNPs on chromosome 4q25 in close proximity to the *PITX2* gene do not directly relate to atrial *PITX2* mRNA concentrations. Nonetheless, given emerging evidence that *PITX2* not only contributes to AF but could be used to predict effectiveness of rhythm control therapy, further investigations into the key co‐factors, regulators and targets of *PITX2* could change the current strategies used to determine the choice of anti‐arrhythmic drugs.

*Pitx2* alters the atrial resting membrane potential and thereby modulates the effectiveness of sodium channel blockers in mice. Reliable methods to identify alterations in *PITX2* expression in humans may help to make an informed choice on anti‐arrhythmic drug therapy.

## Additional information

### Competing interests

L. Fabritz has received institutional research grant support from Deutsche Forschungsgemeinschaft, Medical Research Council, British Heart Foundation and Gilead Inc. P. Kirchhof has received research support from the German Centre for Heart Research, from several drug and device companies active in atrial fibrillation, and has received honoraria from several such companies. F. Syeda, L. Fabritz and P. Kirchhof are listed as inventors on a patent (WO2015/140571) held by the University of Birmingham on genotype‐specific anti‐arrhythmic drug therapy of atrial fibrillation.

### Funding

This work was supported by the European Union (grant agreement No. 633196 (CATCH ME) to P.K. and L.F.), the British Heart Foundation (FS/13/43/30324 to P.K. and L.F.) and the Leducq Foundation to P.K.
